# Podocyte Injury Through Interaction Between *Tlr8* and Its Endogenous Ligand miR-21 in Obstructed and Its Collateral Kidney

**DOI:** 10.3389/fimmu.2020.606488

**Published:** 2021-01-22

**Authors:** Md. Abdul Masum, Osamu Ichii, Yaser Hosny Ali Elewa, Yasuhiro Kon

**Affiliations:** ^1^Laboratory of Anatomy, Department of Basic Veterinary Sciences, Faculty of Veterinary Medicine, Hokkaido University, Sapporo, Japan; ^2^Department of Anatomy, Histology and Physiology, Faculty of Animal Science and Veterinary Medicine, Sher-e-Bangla Agricultural University, Dhaka, Bangladesh; ^3^Laboratory of Agrobiomedical Science, Faculty of Agriculture, Hokkaido University, Sapporo, Japan; ^4^Department of Histology, Faculty of Veterinary Medicine, Zagazig University, Zagazig, Egypt

**Keywords:** chronic kidney disease, obstructive nephropathy, podocyte, TLR8, obstructed and collateral kidney

## Abstract

While chronic kidney disease is prevalent in adults, obstructive nephropathy (ON) has been reported in both young and old patients. In ON, tubulointerstitial lesions (TILs) have been widely investigated, but glomerular lesions (GLs) have been largely neglected. Here, we show a novel mechanism underlying GL development in ON in young and old mice. TILs develop earlier than GLs owing to infiltration of inflammatory cells in the tubulointerstitium, but GLs develop following the activation of *Toll-like receptor 8* (*Tlr8*) even though the absence of inflammatory cells infiltrating the glomerulus. TLR8 and interleukin 1 beta (IL1β) proteins colocalize with reducing podocyte function markers (PFMs), indicating the activation of TLR8 signaling in injured podocytes. Furthermore, glomerular and serum levels of miR-21, an endogenous ligand for *Tlr8*, were higher in the ON mouse model than in the sham control. The glomerular expression of *Tlr8* positively correlates with miR-21 and the downstream cytokines *Il1b* and *Il6* and negatively correlated with PFMs (*Nphs1* and *Synpo*). We also show the colocalization of TLR8 and IL1β proteins with reducing PFMs in both obstructed and collateral kidney of young and old mice. Furthermore, *in vitro* study results revealed higher expression of *Tlr8* and its downstream cytokines in glomeruli from obstructed kidneys following treatment with miR-21 mimic than in the control. In conclusion, the overexpression of *Tlr8* may serve as a plausible mechanism underlying GL development in ON through podocyte injury.

## Introduction

Chronic kidney disease (CKD), a major public health concern, is prevalent in 8%–13% of the general population (1). It is frequent in older people owing to the aging society. Obstructive nephropathy (ON) is of great interest to clinicians, as it has been reported in patients from different age groups. ON is treatable and reversible, unlike other CKDs (2, 3). In children, it occurs in one of 1500 young individuals, and its prevalence is as high as 1%–5% in developed countries (4, 5). In adults, the prevalence of ON ranges from 5 in 1000 to 5 in 10000 individuals. In addition, the prevalence of chronic unilateral ON is approximately 0.5%, while that of acute unilateral and chronic bilateral ON is approximately 0.1% (4–7).

ON can lead to acute kidney injury or CKD, which involves a progressive decrease in renal function and alterations in renal structures lasting for more than 3 months (8, 9). Unilateral ureteral obstruction (UUO) is a widely used strategy to study chronic ON. ON in a UUO kidney is characterized by hypertrophy, hydronephrosis, recruitment of inflammatory cells in the interstitial area, tubular cell death from hypoxia, and collagen deposition in the tubulointerstitium, followed by the development of tubulointerstitial fibrosis (TF) (10).

TF is an important feature of end-stage renal disease and a major determinant of progressive renal injury (11). TF has been extensively investigated in ON models, but data regarding glomerular injury, especially blood-urine barrier (BUB) abnormalities, are scarce (8, 9, 12). Further, studies have investigated tubulointerstitial lesions (TILs) in the collateral kidney in unilateral ON, but glomerular lesions (GLs) have been largely neglected (12). Several reports have revealed the involvement of peritubular capillaries in the progression of TILs in experimental ON (13, 14). However, the glomerulus and glomerular capillary drain into the tubular segments of the nephron and peritubular capillaries, respectively. Therefore, we suggest that glomerulonephritis may also affect the tubulointerstitium. Our previous studies have highlighted the contribution of injury to glomerular intrinsic cells, mainly podocytes, to GL and the subsequent TIL development in an autoimmune disease mouse model due to the overexpression of different Toll-like receptors (TLRs) (15–18).

Glomerular capillary endothelium and podocytes are important gatekeepers comprising BUBs. Between these two epithelia, podocytes are continuously exposed to danger signals, including TLR ligands such as danger-associated molecular patterns (DAMPs) or pathogen-associated molecular patterns (PAMPs), owing to their unique localization in glomeruli (15). Therefore, the interaction between TLRs in podocytes and their endogenous ligands is thought to contribute to podocyte injury in noninfectious conditions. In the present study, we focused on GL development in both the obstructed and collateral kidneys of young and old mice, as ON is frequently found in both young and older humans. We clarified that GL was developed in ON owing to podocyte injury through the overexpression of *Tlr8* in podocytes from both obstructed and collateral kidneys of young and old mice.

## Materials and Methods

### Ethical Statement and Experimental Animal Housing

All experiments using laboratory animals were approved by the Institutional Animal Care and Use Committee of the Faculty of Veterinary Medicine, Hokkaido University (approval no. 16-0124). The authors followed the approved guide for the Care and Use of Laboratory Animals of Hokkaido University, Faculty of Veterinary Medicine (approved by the Association for Assessment and Accreditation of Laboratory Animal Care International). Six-week-old C57BL/6N mice were purchased from Japan SLC Inc. (Hamamatsu, Japan) and maintained in specific pathogen-free conditions under a 1:1 light-dark environment. Experimental animals were provided *ad libitum* food and drinking water.

### Experimental Design

We used both young (9 weeks) and old (12 months) mice. Mice from each age group (n = 4) were subjected to either sham operation (control group) or UUO (2, 7, 11, and 21 days) to establish an ON-model kidney, as described in our previous study (13).

### Sample Preparation

Mice were deeply anesthetized with a mixture of anesthetic agents as previously described (16) and euthanized by cervical dislocation. Blood samples were collected through the femoral artery for serum marker analysis. Both UUO and collateral kidneys were collected and cut into thin slices. Kidney slices were fixed with 10% neutral buffer formalin (NBF), 4% paraformaldehyde (PFA), and 2.5% glutaraldehyde (GTA) for histopathological, immunohistochemical, and electron microscopy studies, respectively.

### Immunohistochemistry and Immunofluorescence

NBF-fixed paraffin blocks were cut at 2 μm thickness and stained with periodic acid Schiff-hematoxylin (PAS-H) to examine renal histopathology. Immunodetection of cellular markers was performed as previously reported (16) for B cells (B220), T cells (CD3), capillary endothelium (CD31), IL1β, macrophages (Iba1), podocytes (podocin and synaptopodin), and TLR8. The staining conditions are listed in **Table 1**.

**Table 1:** Summary of immunostaining conditions.

Parameters	B220	CD3	Iba 1	CD31	IL1 β	Podocin	Synaptopodin	TLR8
**Antigen retrieval**	CB 115°C, 15 min	TB 115°C, 15 min	0.1% pepsin 37°C, 5 min	TB 115°C, 15 min	CB 115°C, 15 min	CB 115°C, 15 min	CB 115°C, 15 min	CB 115°C, 15 min
**Blocking**	10% NGS	10% NGS	10% NGS	5% NDS	5% NDS	5% NDS	5% NDS	5% NDS
**Primary antibody**	Rat polyclonal antibodies (Cedarlane, ON, Canada) 1:1000	Rabbit polyclonal antibodies (Nichirei, Tokyo, Japan) 1:200	Rabbit polyclonal antibodies (Wako, Tokyo, Japan) 1:2000	Rabbit polyclonal antibodies (Abcam, Cambridge, UK) 1:150	Goat monoclonal antibodies (R and D system, Minneapolis, USA) 1:200	Rabbit polyclonal antibodies (1:800; IBL, Gunma, Japan)	Mouse monoclonal antibodies (1:100; Fitzgerald, MA, USA)	Rabbit polyclonal antibodies (Abcam, Cambridge, UK) 1:700
**Secondary antibody**	Goat anti-rat IgG (Caltag Medsystems, Buckingham, UK) 1:100 (Biotinylated)	Goat anti-rabbit (SABPO kit, Nichirei, Tokyo, Japan) 1:100 (Biotinylated)	Goat anti-rabbit (SABPO kit, Nichirei, Tokyo, Japan) 1:100 (Biotinylated)	Alexa Fluor 546-labeled donkey anti-rabbit IgG antibodies (1:500; Life Technologies)	Alexa Fluor 488-labeled donkey anti-goat IgG antibodies (1:500; Life Technologies)	Alexa Fluor 546-labeled donkey anti-rabbit IgG antibodies (1:500; Life Technologies)	Alexa Fluor 546-labeled donkey anti-mouse IgG antibodies (1:500; Life Technologies)	Alexa Fluor 488-labeled donkey anti-rabbit IgG antibodies (1:500; Life Technologies)

CB, Citrate buffer; TB, Tris buffer; NGS, Normal goat serum; NDS, Normal donkey serum.

### Histoplanimetry

PAS-H and immunostained kidney sections were converted to virtual slides using Nano Zoomer 2.0 RS (Hamamatsu Photonics Co., Ltd.; Hamamatsu, Japan). Positive cells were counted from randomly selected 20 glomeruli or 20 foci from the tubulointerstitium at 400x magnification using NDP.view2 software (Hamamatsu Photonics Co., Ltd.). Images of 20 glomeruli from PAS-H-stained sections were also captured at 400x from each mouse using an All-in-One Fluorescence Microscope BZ-X710 (Keyence, Osaka, Japan). The glomerular mesangial area and size were measured from captured images using a BZ-X Analyzer (Keyence).

### Isolation of Glomerulus for RNA Extraction and Culture

Glomeruli from sham-operated and UUO kidneys were isolated as previously described (16). Briefly, mice were deeply anesthetized and perfused through the left ventricle with 40 mL of Hank’s balanced salt solution (HBSS) containing Dynabeads (8 × 10^7^; Life Technologies, Carlsbad, CA, USA). The kidney was excised and chopped into small pieces. The cells were then digested with collagenase A (1 mg/mL; Roche, Basel, Switzerland) and deoxyribonuclease I (100 U/ml; Life Technologies) in HBSS at 37°C for 30 min. The suspension containing digested tissue was gently pressed through a 100-μm cell strainer (BD Falcon, Franklin Lakes, NJ, USA) using a flattened pestle. The resulting cell suspension was centrifuged at 200 ×*g* for 5 min, and the cell pellet was resuspended in 2 ml of HBSS. Glomeruli containing Dynabeads were collected using a magnetic particle concentrator (Life Technologies) and used for RNA isolation.

### Reverse Transcription and Real-Time PCR

Total RNA was isolated from glomeruli using a RNeasy kit (Qiagen, Hilden, Germany). cDNA was synthesized from total RNA by reverse transcription using the ReverTra Ace reverse transcriptase enzyme (Toyobo, Osaka, Japan) and random dT primers (Promega). The cDNA was used in real-time PCR with Brilliant III SYBR Green QPCR master mix and Mx3000P (Agilent Technologies, La Jolla, CA, USA). Glomerular gene expression was normalized to the expression of *Actb*. The primer pairs used are shown in **Table 2**.

**Table 2:** Details of primers used in this study.

Gene name (Accession number)	Primer sequence (5^’^-3^’^)	Product size	Application
*Actb* (NM007393)	F: TGTTACCAACTGGGACGACA R: GGGGTGTTGAAGGTCTCAAA	165	Real-time PCR
*Tlr1* (NM_030682)	F: GTGAATGCAGTTGGTGAAGAAC R: ATGGCCATAGACATTCCTGAG	125	Real-time PCR
*Tlr2* (NM_011905)	F: GAGCATCCGAATTGCATCA R: CACATGACAGAGACTCCTGAGC	163	Real-time PCR
*Tlr3* (NM_126166)	F: GATACAGGGATTGCACCCATA R: GCATTGGTTTGTGGAAGACAC	122	Real-time PCR
*Tlr4* (NM_021297)	F: TTCAGAACTTCAGTGGCTGGA R: CTGGATAGGGTTTCCTGTCAGT	115	Real-time PCR
*Tlr5* (NM_016928)	F: ATGCCAGACACATCTGTGAGA R: ATCCTGCCGTCTGAAGAACA	177	Real-time PCR
*Tlr6* (NM_011604)	F: ATGGTACCGTCAGTGCTGGA R: TCTGTCTTGGCTCATGTTGC	104	Real-time PCR
*Tlr8* (NM_133212)	F: GTTATGTTGGCTGCTCTGGTTCAC R: TCACTCTCTTCAAGGTGGTAGC	203	Real-time PCR
*Tlr9* (NM_031178)	F: GAATCCTCCATCTCCCAACA R: GGGTACAGACTTCAGGAACAGC	181	Real-time PCR
*Nphs1* (NM_019459)	F: ACCTGTATGACGAGGTGGAGAG R: TCGTGAAGAGTCTCACACCAG	218	Real-time PCR
*Synpo* (NM_177340.2)	F: CATCGGACCTTCTTCCTGTG R: TCGGAGTCTGTGGGTGAG	90	Real-time PCR
*Il1b* (NM008361)	F: AAGGAGAACCAAGCAACGAC R: AACTCTGCAGACTCAAACTCCAC	208	Real-time PCR
*Ifng* (NM008337.3)	F: CCTTTGGACCCTCTGACTTG R: TTCCACATCTATGCCACTTGAG	201	Real-time PCR
*Il6* (NM031168)	F: TGTATGAACAACGATGATGCAC R: TGGTACTCCAGAAGACCAGAGG	137	Real-time PCR
*Tgfb* (NM011577)	F: AGCCTGGACACACAGTACAGC R: CGACCCACGTAGTAGACGATG	125	Real-time PCR
*Tnf* (NM013693)	F: CGAGTGACAAGCCTGTAGCC R: GAGAACCTGGGAGTAGACAAGG	167	Real-time PCR

F, Forward; R, Reverse.

### Reverse Transcription and TaqMan-Based Real-Time PCR

Total RNA, including microRNA (miRNA) in the isolated glomeruli, was isolated using an miRNeasy kit (Qiagen). miRNA-specific stem-loop RT primers, reverse transcriptase, reverse transcription buffer, dNTPs, and RNase inhibitor were used to reverse transcribe total RNA according to the manufacturer’s instructions (Applied Biosystems, Foster City, CA, USA). Real-time PCR was performed with the resulting cDNA using miR-21-specific TaqMan primers and specific probes (Applied Biosystems), TaqMan Universal PCR Master Mix (Applied Biosystems), and Mx3000P (Agilent Technologies).

### *In Vitro* Treatment of Isolated Glomeruli With Mimics

Glomeruli were isolated from sham-operated and UUO kidneys after 11 days of obstruction. Roswell Park Memorial Institute-1640 (RPMI-1640) medium (Fujifilm Wako, Japan) containing 300 glomeruli was distributed in each well of a 96-well culture plate (TPP, Trasadingen, Switzerland) and treated with PBS, negative control, and miR-21 mimic (UAG CUU AUC AGA CUG AUG UUG A) (Bioneer, Daejeon, South Korea) at 1 pmol/µL for 4 h at 37°C in 5% CO_2_. The expression of *Tlr8* and its downstream cytokines was measured as described in the previous section.

### Scanning Electron Microscopy

For routine scanning electron microscopy (SEM), small kidney slices were fixed with 2.5% GTA for 4 h and post-fixed with 1% osmium tetroxide for 1 h, followed by treatment with 1% tannic acid for 1 h. The samples were then fixed with 1% osmium tetroxide for 1 h and treated with 0.5% and 1% tannic acid for 10 min and 1 h, respectively. Kidney slices were dehydrated with ascending grades of alcohol, transferred into 3-methylbutyl acetate, and finally dried with a HCP-2 critical point dryer (Hitachi, Tokyo, Japan). The specimens were subjected to ion sputtering and then examined under an S-4100 scanning electron microscope at an accelerated voltage of 12 kV.

### Statistical Analysis

The values are expressed as the mean ± standard error (s.e.). The results were statistically analyzed using a nonparametric Mann–Whitney U-test (P < 0.05). The Kruskal-Wallis test was used to compare three or more populations, and multiple comparisons were performed using Scheffe’s method once significant differences were observed (P < 0.05). The correlation between the two parameters was analyzed using Spearman’s rank correlation coefficient test (P < 0.05).

## Results

### TILs and GLs in the Obstructed Kidneys of Young Mice

**Figure 1:**
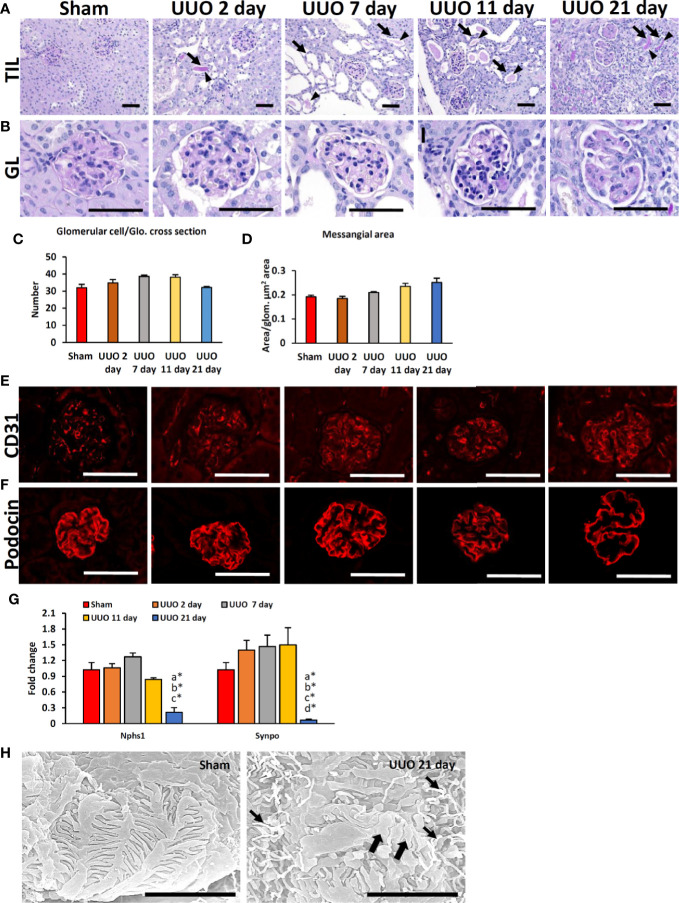
Renal lesions and blood-urine barrier injury in UUO kidneys of young mice at different time periods. **(A)** The sham-operated kidneys showed normal tubules. TIL was characterized by dilatation of tubules (arrows); urinary cast (arrowhead) was observed in 2-, 7-, 11-, and 21-day UUO kidneys. **(B)** The glomerulus was normal in sham-operated, and 2-day UUO. An increasing tendency of glomerular cell was noted in UUO kidneys until 11 days following obstruction. PAS-positive material deposition tendency was noted in UUO kidney glomeruli from 7 days following obstruction. Periodic acid Schiff hematoxylin. Bars = 50 µm. **(C)** Glomerular cell number per glomeruli in sham, 2-, 7-, 11- and 21-day UUO kidneys. **(D)** Mesangial area in glomerulus of sham, 2-, 7-, 11-, and 21-day UUO kidneys. **(E)** CD31-positive glomerular capillary endothelia were clearly visible in sham and obstructed kidneys. **(F)** Podocin localization was normal in sham, 2-, 7-, 11- and 21-day UUO kidneys. Podocin protein expression was lost at the center of the glomeruli of UUO kidneys at 21 days after obstruction. Immunofluorescence, Bars = 50 µm. **(G)** Relative mRNA expression of *Nphs1* and *Synpo* glomerulus of sham, 2-, 7-, 11- and 21-day UUO kidneys. The expression levels were normalized to the level of *Actb*. Values are expressed as mean ± s.e. Significant differences from the other groups are indicated by * (*P < 0.05, Kruskal-Wallis test followed by Scheffe’s method). n = 4. a, b, c, d, and e denote sham, 2-, 7-, 11- and 21-day UUO kidneys, respectively. **(H)** PFPs were normal in sham-operated kidneys. PFP effacement (thick arrow) and a microvillous-like structure (thin arrow) were observed in UUO kidney. Scanning electron microscopy, Bars = 4 µm. GL, Glomerular lesion; TIL, Tubulointerstitial lesion; UUO, Unilateral ureteral obstruction; Glo., glomeruli; PFP, Podocyte foot process.

We first examined TILs and GLs in sham-operated and UUO kidneys of young mice subjected to obstruction for different time periods. While the sham-operated kidneys showed normal tubulointerstitium (**Figure 1A**), the obstructed kidneys after 2 days of UUO showed TILs and were characterized by the dilatation of tubules; some tubules also contained urinary casts. Such lesions increased with the advancement of obstruction (**Figure 1A**). GLs, mainly glomerulosclerosis, were observed in the late stage at 21 days following UUO and characterized by the deposition of periodic acid-Schiff (PAS)-positive materials. The glomerular structure was normal in both sham and early UUO kidneys (2, 7, and 11 days) (**Figure 1B**). The number of glomerular cells were tended to increase until day-11 following obstruction and tended to decrease at day-21 in the UUO kidneys (**Figure 1C**). Glomerular mesangial accumulation was tended to increase from 7 days following obstruction (**Figure 1D**).

### BUB injury in the Obstructed Kidney

To analyze the integrity of the BUB in the glomerular capillary endothelium and podocytes, we examined the expression of CD31 and podocin. The CD31-positive capillary endothelium was normal in both sham-operated and UUO kidneys at different time points following obstruction (**Figure 1E**). The expression of the podocyte function marker (PFM), podocin, was observed in the sham and 2-, 7-, and 11-day UUO kidneys (**Figure 1F**) but was absent at the center of the glomeruli of UUO kidneys at 21 days following obstruction (**Figure 1F**). Polymerase chain reaction revealed a significant decrease in PFMs (*Nphs1* and *Synpo*) in UUO kidneys 21 days following obstruction (**Figure 1G**). Moreover, SEM analysis revealed normal podocyte foot processes (PFPs) in sham-operated kidneys; however, PFP effacement and microvillous-like structures were reported in UUO kidneys 21 days after obstruction (**Figure 1H**). Together, these results suggest the occurrence of podocyte injury in the obstructed kidney 21 days after obstruction.

### Infiltrating Immune Cells Are Absent in the Obstructed Kidney

**Figure 2:**
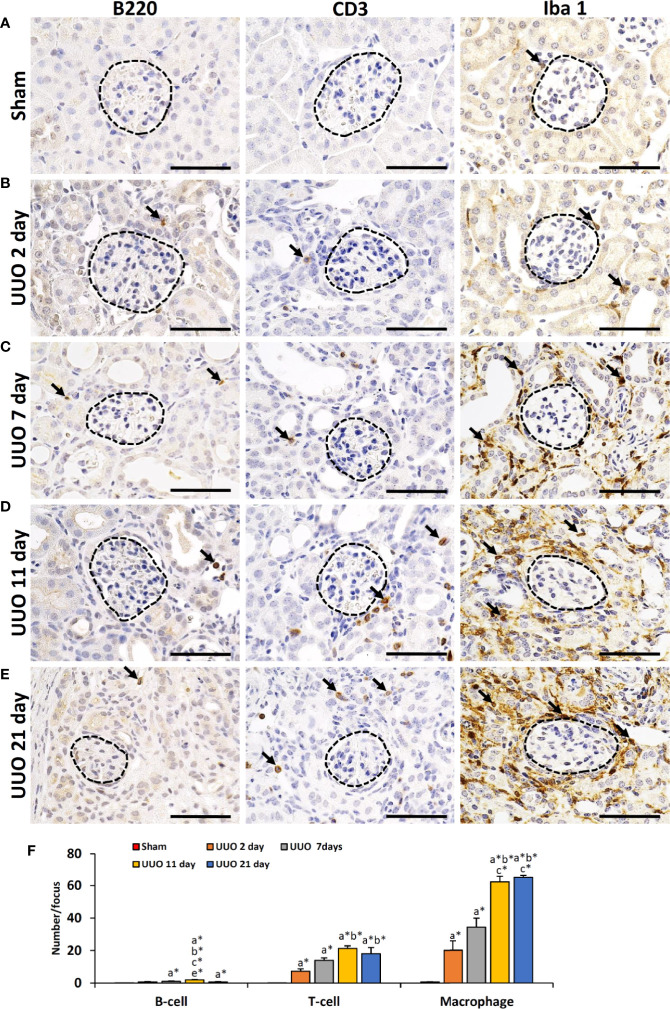
Infiltration of immune cells in TILs and GLs of UUO kidneys of young mice at different time periods. **(A)** B220^+^ B cells, CD3^+^ T cells, and Iba1^+^ macrophages (arrow) in sham-operated mouse kidneys. **(B)** B220^+^ B cells, CD3^+^ T cells, and Iba1^+^ macrophages (arrow) in TILs and GLs of UUO 2-day kidneys. **(C)** B220^+^ B cells, CD3^+^ T cells, and Iba1^+^ macrophages (arrow) in TILs and GLs of UUO 7-day kidneys. **(D)** B220^+^ B cells, CD3^+^ T cells, and Iba1^+^ macrophages (arrow) in TILs and GLs of UUO 11-day kidneys. **(E)** B220^+^ B cells, CD3^+^ T cells, and Iba1^+^ macrophages (arrow) in TILs and GLs of UUO 21-day kidneys. **(F)** Number of B220^+^ B cells, CD3^+^ T cells, and Iba1^+^ macrophages per focus in sham, 2-, 7-, 11-, and 21-day UUO kidneys. Values are expressed as mean ± s.e. Significant differences from the other groups are indicated by * (*P < 0.05, Kruskal-Wallis test followed by Scheffe’s method). n = 4. a, b, c, d, and e denote sham, 2-, 7-, 11-, and 21-day UUO kidneys, respectively. TIL, Tubulointerstitial lesion; GL, Glomerular lesion; UUO, Unilateral ureteral obstruction.

We have previously shown that podocyte injury correlates with infiltrating immune cells in the glomerulus (13, 16, 18). Therefore, we examined the infiltration of B- and T-cells as well as macrophages in TILs and GLs of sham-operated and UUO kidneys (**Figure 2**). Numerous infiltrating B-, T-cells and macrophages were observed in the TILs of UUO kidneys on all days following obstruction, but they were almost absent or few in the glomeruli of both sham and UUO kidneys (**Figures 2A–E**). The number of B-, T-cells and macrophages were very few in glomerulus (data not shown) but significantly higher in the tubulointerstitium of 2-, 7-, 11-, and 21-day kidneys compared to that of the sham kidney (**Figure 2F**). Therefore, infiltrating immune cells may contribute to the development of TILs in the obstructed kidney. However, other factors may be related to GL development.

### Expression of Different Members of the TLR Family and Downstream Cytokines in Sham-Operated and Obstructed Kidneys of Young Mice

**Figure 3:**
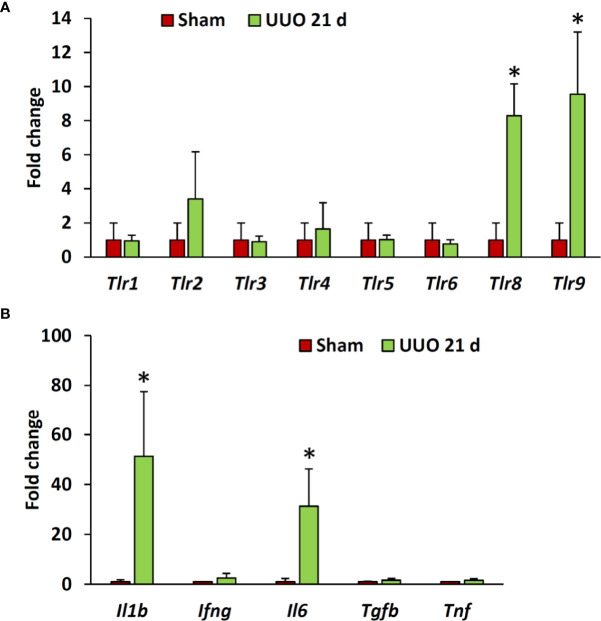
Relative mRNA expression of *Tlr* family members and their downstream cytokines in the glomeruli isolated from sham-operated and UUO kidneys of young mice at day 21. **(A)** Relative mRNA expression of *Tlr* family members in glomeruli isolated from sham-operated and UUO kidneys at day 21 following obstruction. **(B)** Relative mRNA expression of downstream cytokines of *Tlr* family members in glomeruli from sham-operated and UUO kidneys at day 21 following obstruction. The expression levels were normalized to the level of *Actb*. Values are expressed as mean ± s.e. *significantly different from control sham-operated kidneys (Mann-Whitney U-test, P < 0.05); qPCR, n = 4. *Tlr*, *Toll-like receptor*; *Il1b*, *Interleukin 1-beta*; *Ifng*, *Interferon gamma*; *IL6*, *Interleukin 6*; *Tgfb*, *Transforming growth factor beta*; *Tnf*, *Tumor necrosis factor*; UUO, Unilateral ureteral obstruction.

Previous studies have revealed higher expression of different TLRs in podocytes of diseased kidneys (15, 16, 19, 20). Interestingly, our data revealed significantly higher expression of *Tlr8* and *Tlr9* in the glomeruli isolated from the UUO 21-day group than in the glomeruli of the sham group (**Figure 3A**). Furthermore, we examined the expression of downstream cytokines of the TLR family members in the glomeruli from sham and UUO 21-day groups and found a significant upregulation in the expression of the genes encoding both interleukin 1 beta (*Il1b*) and interleukin 6 (*Il6*) in the UUO group compared to that in the sham group (**Figure 3B**).

### TLR8 Co-Localized With PFM

**Figure 4:**
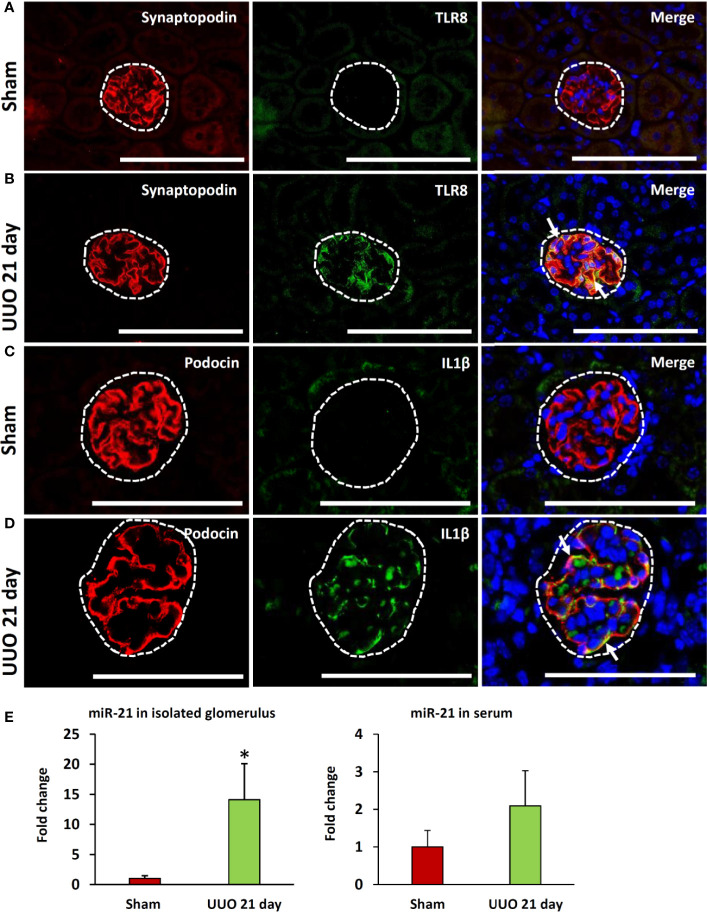
Colocalization of PFMs with TLR8 and expression of TLR8 ligand in ON kidneys of young mice at day 21 following obstruction. **(A)** Localization of PFM (synaptopodin) and TLR8 in the glomerulus (dashed area) of sham-operated mice. **(B)** Colocalization of PFM (synaptopodin) and TLR8 in the glomerulus (dashed area) of UUO kidneys. **(C)** Localization of PFM (podocin) and IL1β in the glomerulus (dashed area) of sham-operated mice. **(D)** Colocalization of PFM (podocin) and IL1β in the glomerulus (dashed area) of UUO kidneys. Immunofluorescence, Bars = 100 µm. **(E)** The relative expression of miR-21 in the glomeruli and serum from sham-operated and UUO kidneys at 21 days. Values are expressed as mean ± s.e. Data are presented as fold increase versus sham-operated kidneys. *significantly different from control sham-operated kidneys (Mann-Whitney U-test, P < 0.05); n = 4. TLR8, Toll-like receptor 8; UUO, unilateral ureteral obstruction; PFM, podocyte function marker; ON, obstructive nephropathy.

As the expression of *Tlr8* and *Tlr9* was high in the glomeruli isolated from the obstructed kidney, we examined their localization by immunofluorescence staining. TLR8 protein was not detected in sham-operated kidneys (**Figure 4A**) but was colocalized with the PFM synaptopodin in the UUO kidney (**Figure 4B**). Furthermore, we failed to detect TLR9 protein expression in the kidney from either sham-operated or UUO mice (data not shown).

### IL1β Production From Podocytes in the Obstructed Kidney of Young Mice

As shown in **Figure 3B**, the expression of glomerular *Il1b* (a downstream cytokine of the TLR family) was significantly higher in the UUO kidney at 21 days than that in the sham kidney. Therefore, we examined the source of IL1β in the glomeruli of UUO kidneys by immunofluorescence staining. IL1β protein expression was not detected in the glomeruli of sham kidneys but was observed in the glomeruli of UUO kidneys (**Figures 4C, D**). Furthermore, IL1β colocalized with PFM podocin (**Figure 4D**).

### Increased Level of the Putative Endogenous *Tlr8* Ligand in ON Model Mice

A previous study showed that miR-21 serves as a ligand for TLR8 (21). Therefore, we compared both the glomerular and serum levels of miR-21 between the sham and UUO 21-day groups. Interestingly, the UUO 21-day group showed significantly higher levels of glomerular miR-21 than that in sham group (P < 0.05), however, an increasing tendency of serum miR-21 was observed in the former group than the later (P = 0.06) (**Figure 4E**).

### Correlation of *Tlr8* Expression With Its Ligand miR-21 and PFMs in the Obstructed Kidney

As shown in **Table 3**, we observed a significant positive correlation between the glomerular expression of *Tlr8* mRNA and its endogenous ligand (miR-21) as well as the downstream cytokines *Il1b* and *Il6*. On the other hand, a negative correlation was evident between *Tlr8* mRNA and the PFMs *Nphs1* and *Synpo*.

**Table 3:** Correlation of glomerular *Tlr8* expression with its ligand, downstream cytokines, and podocyte function markers.

Parameters	*Glo. Tlr8*		*Glo. miR-21*		*Serum miR-21*		*Glo. Il1b*		*Glo. Il6*		*Glo. Nphs1*		*Glo. Synpo*	
	*ρ*	P	*ρ*	P	*ρ*	P	*ρ*	P	*ρ*	P	*ρ*	P	*ρ*	P
***Glo. Tlr8***	1.00	–	0.78^*^	<0.05	0.28	0.53	0.77^*^	<0.05	0.82^*^	<0.05	−0.75	0.052	−0.75	0.052
***Glo. Il1b***	0.77^*^	<0.05	0.70	0.07	0.30	0.50	1.00	–	0.88^**^	<0.01	−0.81^*^	<0.05	−0.95^**^	<0.01
***Glo. Il6***	0.82^*^	<0.05	0.89^**^	<0.01	0.21	0.64	0.88^**^	<0.01	1.00	–	−0.96^**^	<0.01	−0.96^**^	<0.01

^*^P < 0.05 and ^**^P < 0.01, Spearman’s rank correlation coefficient, N = 8. Glo., Glomerular; Tlr8, Toll-like receptor 8; Il1b, Interleukin 1 beta; Il6, Interleukin 6.

### *In Vitro* Stimulation of Glomeruli With miR-21 Mimic Activates the Expression of *Tlr8* and Its Downstream Cytokines

**Figure 5:**
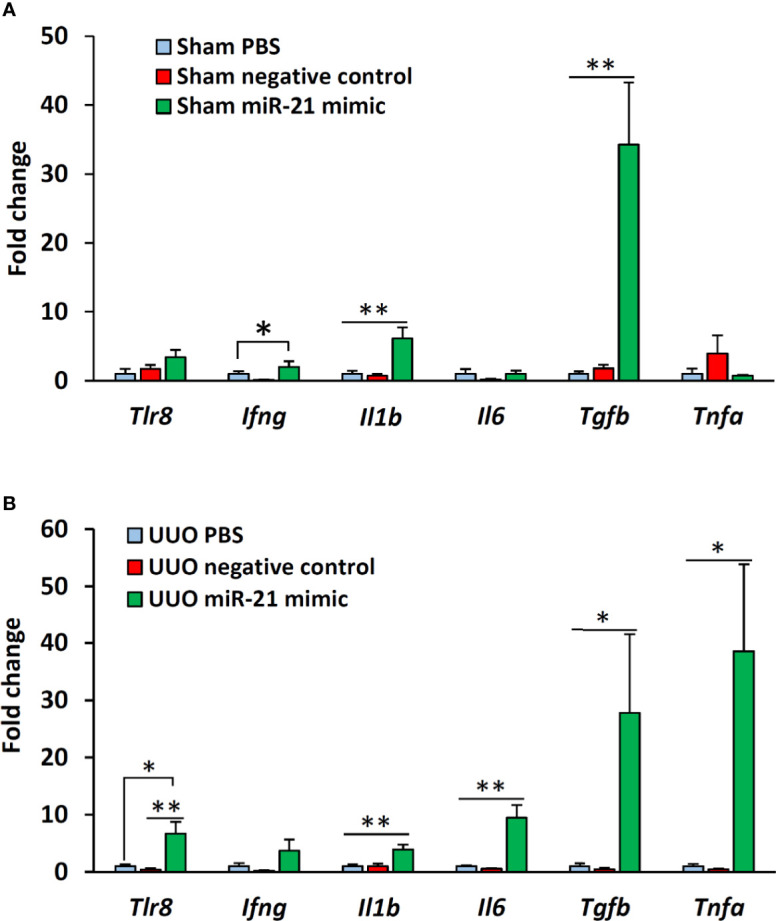
*In vitro* expression of *Tlr8* and its downstream cytokines after treatment with mimics. **(A)** Expression of *Tlr8* and its downstream cytokines in the glomeruli isolated from sham control mice after treatment with PBS, negative control, and miR-21 mimic for 4 h. **(B)** Expression of *Tlr8* and its downstream cytokines in the glomeruli from obstructed kidneys after treatment with PBS, negative control, and miR-21 mimic for 4 h. The values are the mean ± s.e. Significant difference from the other is indicated by * (*P < 0.05, **P < 01, Kruskal-Wallis test followed by Scheffe’s method). n = 4.

The expression of *Tlr8* was tended to increase (P = 0.06) but its downstream cytokines (*Ifng* and *Il1b*) were significantly (P < 0.05 and 0.01) increased in the glomeruli of sham mice following treatment with miR-21 mimic as compared to that in the glomeruli of mice treated with PBS and negative control mimic (**Figure 5A**). However, no significant correlation was observed between the expression of *Tlr8* and its downstream cytokines in the glomeruli of sham mice following treatment with mimics (**Table 4**). The expression of *Tlr8* and its downstream cytokines was significantly higher in the glomeruli of UUO kidneys, following treatment with miR-21 mimic, than those in the glomeruli of UUO kidneys treated with PBS and negative control mimic (**Figure 5B**). A significant correlation was detected between the expression of *Tlr8* and its downstream cytokines in the glomeruli of UUO kidneys following treatment with mimics, indicative of the higher activation of the *Tlr8*-mediated nuclear factor kappa B (NF-κB) pathway and secretion of proinflammatory cytokines in the obstructed kidney (**Table 4**).

**Table 4:** Correlation of *Tlr8* wits its downstream cytokines in mimics treated glomerulus obtained from sham and obstructed kidney.

Parameter	*Ifng*		*Il1b*		*Il6*		*Tnf*		*Tgfb*	
	*ρ*	P	*ρ*	P	*ρ*	P	*ρ*	P	*ρ*	P
Glo. *Tlr8* expression in sham kidney treated with mimic	0.49	0.10	0.53	0.07	0.43	0.15	-0.28	0.36	0.46	0.13
Glo. *Tlr8* expression in obstructed kidney treated with mimic	0.77**	<0.01	0.88**	<0.01	0.71**	<0.01	0.79**	<0.01	0.67*	0.016

^*^P < 0.05 and ^**^P < 0.01, Spearman’s rank correlation coefficient, N = 12. Tlr8, Toll-like receptor 8; Glo., Glomerular; Ifng, Interferon gamma; Il1b, Interleukin 1 beta; Il6, Interleukin 6; Tnf, Tumor necrosis factor; Tgfb, Transforming growth factor beta.

### TLR8 Localization and Podocyte Injury in the Collateral Kidneys of Young Mice

**Figure 6:**
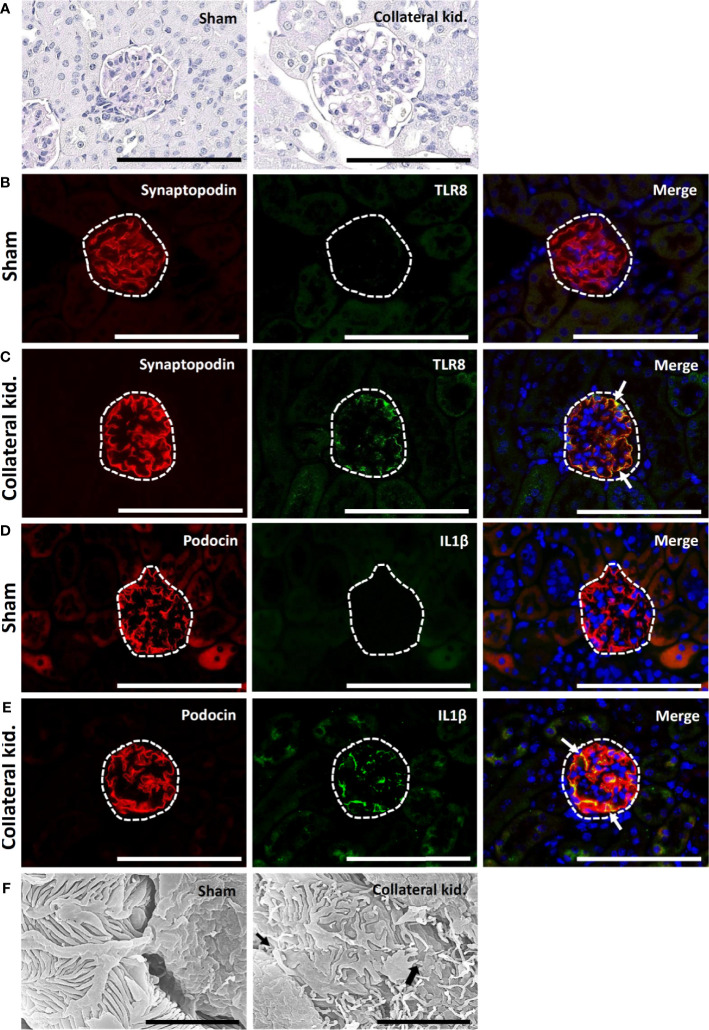
Glomerular lesion, colocalization of PFM with TLR8, and podocyte injury in collateral kidneys of young mice subjected to UUO. **(A)** Sham-operated mice showed normal glomeruli. The collateral kidney of younger mice that underwent UUO showed glomerular hypertrophy with an increase in the population of glomerular cells and dilation of glomerular capillaries. PAS-H stain, Bars = 100 µm. **(B)** Localization of PFM (synaptopodin) and TLR8 in the glomerulus (dashed area) of sham-operated mice. **(C)** Colocalization of PFM (synaptopodin) and TLR8 in the glomerulus (dashed area) of the collateral kidneys. **(D)** Localization of PFM (podocin) and IL1β in the glomerulus (dashed area) of sham-operated mice. **(E)** Colocalization of PFM (podocin) and IL1β in the glomerulus (dashed area) of the collateral kidneys. Immunofluorescence, Bars = 100 µm. **(F)** PFPs were normal in sham-operated kidneys. Disorganized PFPs (thick arrow) and a microvillous-like structure (thin arrow) were observed in the collateral kidneys of younger mice that underwent UUO. Scanning electron microscopy, Bars = 4 µm. PAS-H, Periodic acid-Schiff-hematoxylin; Kid., Kidney; UUO, Unilateral ureteral obstruction; TLR8, Toll-like receptor 8; PFM, Podocyte function marker; PFP, Podocyte foot process.

The sham-operated kidneys showed normal glomerulus, whereas the collateral kidneys had glomerular hypertrophy, increased glomerular cell number, and dilatation of glomerular capillaries (**Figure 6A**). The glomeruli from sham-operated kidneys were positive for synaptopodin expression and lacked TLR8 expression (**Figure 6B**). On the other hand, the collateral kidneys lost synaptopodin expression at the center of glomeruli but showed TLR8 expression along the glomerular rete (**Figure 6C**). Moreover, TLR8 protein was co-localized with synaptopodin (**Figure 6C**). Sham-operated kidneys showed normal expression of podocin but no expression of IL1β in glomeruli (**Figure 6D**). However, the glomeruli within the collateral kidney lost podocin expression at the center but had IL1β expression along the glomerular rete (**Figure 6E**). Furthermore, the IL1β protein was colocalized with podocin (**Figure 6E**). SEM analysis revealed normal PFPs in sham-operated mouse kidneys, but PFP effacement and microvillous-like structures were visible in the collateral kidneys (**Figure 6F**).

### TLR8 Localization and Podocyte Injury in the Obstructed and Collateral Kidneys of Aged Mice

**Figure 7:**
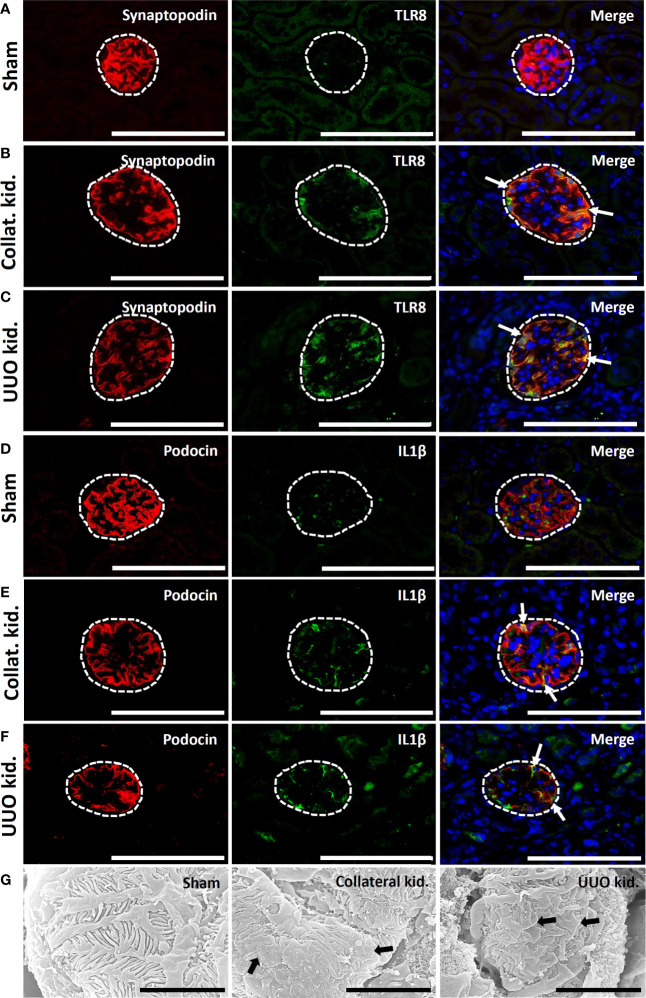
Colocalization of PFM with TLR8 protein and podocyte injury in collateral and obstructed kidneys of older mice. **(A)** Localization of PFM (synaptopodin) and TLR8 in the glomerulus (dashed area) of sham-operated mice. **(B)** Colocalization of PFM (synaptopodin) and TLR8 in the glomerulus (dashed area) of the collateral kidney. **(C)** Colocalization of PFM (synaptopodin) and TLR8 in the glomerulus (dashed area) of UUO kidneys. **(D)** Localization of PFM (podocin) and IL1β in the glomerulus (dashed area) of sham-operated mice. **(E)** Colocalization of PFM (podocin) and IL1β in the glomerulus (dashed area) of the collateral kidney. **(F)** Colocalization of PFM (podocin) and IL1β in the glomerulus (dashed area) of UUO kidneys. Immunofluorescence, Bars = 100 µm. **(G)** The PFPs were normal in sham-operated kidneys. PFP effacement (thick arrow) was observed in the collateral and UUO kidneys of older mice on day 21 following obstruction. Scanning electron microscopy, Bars = 4 µm. Collat. kid., Collateral kidney; UUO, Unilateral ureteral obstruction; PFM, Podocyte function marker; PFP, Podocyte foot process; TLR8, Toll-like receptor 8.

Sham-operated kidneys showed normal expression of synaptopodin but revealed no staining for TLR8 protein within their glomeruli (**Figure 7A**). Interestingly, both collateral and UUO kidneys showed loss of synaptopodin expression at the center of glomeruli but maintained TLR8 expression along the glomerular rete (**Figures 7B, C**). TLR8 protein was colocalized with synaptopodin in the collateral and UUO kidneys (**Figures 7B, C**). Sham-operated kidneys similarly showed normal podocin expression but had no IL1β expression in their glomeruli (**Figure 7D**). On the other hand, both collateral and UUO kidneys lost podocin expression at the center of glomeruli but showed IL1β expression along the glomerular rete (**Figures 7E, F**). Positive staining was found to be colocalized with podocin in the glomeruli of the collateral and UUO kidneys (**Figures 7E, F**). SEM examination revealed normal PFPs in sham-operated mouse kidneys, PFP effacement, and microvillous-like structures in both the collateral and UUO kidneys of old mice (**Figure 7G**).

## Discussion

ON prevalence is common in infants as well as in aged individuals (4–7). Hence, we subjected young and old mice to UUO to mimic the ON condition from individuals of different age groups and examined TILs and GLs. In the obstructed kidney, the progression of TILs was evident at an earlier stage of obstruction (2 days); however, the progression of GLs along with podocyte injury was observed at a later stage. The collateral kidney showed GLs and podocyte injury at the same time. This observation indicates that podocyte injury occurs both in the obstructed and collateral kidneys, although the initial insult may vary.

Our previous studies have highlighted the correlation between podocyte injury and infiltration of immune cells in the glomerulus (13, 16, 18). However, immune cell infiltration into the glomeruli of either sham or obstructed kidneys was not observed in the present study. Therefore, we considered the involvement of other factors in podocyte injury in the obstructed kidney. We hypothesized that danger signals (DAMPs or PAMPs), either of circulatory origin or arising from damaged tubular epithelium, may contribute to podocyte injury in ON, owing to its unique location in the glomerulus. Importantly, the interaction between DAMPs and TLRs has been shown to play an important role in the pathogenesis of noninfectious diseases (15).

Different members of the TLR family are expressed on the cell plasma membrane or intracellular vesicles (22) and are characterized as innate immune sensors that efficiently recognize DAMPs or PAMPs. Upon activation by corresponding DAMPs or PAMPs, TLRs enhanced the expression of downstream cytokines through the NF-κB pathway and induced the host defense system (22). Moreover, DAMPs relay the presence of tissue injury to immune cells or local intrinsic cells, and consequently aggravate tissue damage (23–26). Importantly, all TLRs activate the NF-κB pathway that controls the expression of an array of inflammatory cytokine genes (27). In addition, activation of TLRs signal their downstream pathways to activate NF-κB, which is responsible for inflammation and linked to the pathogenesis of damaged tissues (28). Shichita et al. revealed the pathological interactions between endogenous ligands and TLR2 or TLR4, which contribute to ischemic brain injury (25). Previous studies have also demonstrated the ability of the members of the TLR family to induce TILs in the kidney (TLR2, 4, 5, 7, and 11) and GLs (TLR1–6, 8, and 9) (17, 29–33). In the present study, we found podocyte injury in obstructed and collateral kidneys and clarified the roles of different members of the TLR family that may contribute to podocyte injury (15, 16, 19, 20). As we hypothesized, the glomerular expression of *Tlr8* and *Tlr9* was higher in the glomeruli isolated from the UUO kidney than in those from sham-operated mice. Moreover, the glomerular expression of inflammatory cytokines related to the NF-κB pathway, including *Il1b* and *Il6*, was higher in the UUO kidney at 21 days following obstruction. Therefore, we concluded that the TLR-mediated NF-κB pathway plays an important role in the pathogenesis of GL in the obstructive kidney.

Unlike other members of the TLR family, TLR8 and TLR9 are detected in the endosomes of cells. TLR8 recognizes single-stranded RNAs and short double-stranded RNAs from microorganisms and activates the production of several NF-κB-mediated cytokines (22). On the other hand, TLR9 recognizes double-stranded DNAs and activates the NF-κB pathway to produce downstream cytokines (16, 34). In this study, we only recognized the colocalization of TLR8 protein along with the PFM synaptopodin in glomeruli. Our previous studies have also shown that both TLR8 and TLR9 co-localize with PFMs and activate NF-κB-mediated inflammatory cytokine production to induce podocyte injury (15, 16). Moreover, other studies have shown the pathological correlation between TLR-mediated pathways and podocyte injury *in vitro* (34, 35). Banas et al. revealed the interaction of TLR4 in podocytes with the immune system during GL development (35). Thus, podocytes may contribute to immune surveillance through TLR-mediated pathways. In this study, we showed both TLR8 gene expression and protein localization in the podocytes of the obstructed kidney as well as the higher expression of the downstream cytokines in the glomerulus. Therefore, we conclude that TLR8-mediated pathways contribute to GL development through podocyte injury in the obstructed kidney.

A previous study showed that miR-21, an endogenous ligand of TLR8, can reach and bind to TLR8 in cellular endosomes, wherein it can induce the TLR8-mediated activation of the NF-κB pathway and secretion of proinflammatory cytokines (21). In the present study, we found significantly higher levels of glomerular miR-21 as well as elevated levels of serum miR-21 in the UUO kidney than those in sham controls and demonstrated its correlation with the glomerular expression of *Tlr8*. Interestingly, the glomeruli from UUO kidneys treated with miR-21 mimic showed higher expression of *Tlr8* and its downstream cytokines compared to those in the glomeruli from sham mice treated with miR-21 mimic, indicating increased availability of endogenous miR-21 from damaged tissues in the obstructed kidney and activation of the overexpression of *Tlr8* in UUO kidneys compare to that of sham. Together, we conclude that a higher volume of miR-21 in the obstructed kidney reaches the endosomes in podocytes and induces *Tlr8*-mediated activation of the NF-κB pathway and secretion of proinflammatory cytokines. These cytokines, in turn, participate in GL development through podocyte injury.

IL1β is one of the important downstream cytokines of the NF-κB-pathway, mainly produced by podocytes in the glomerulus under disease conditions. Previous studies have shown that IL1β induces podocyte injury by reducing PFMs (36, 37). In the present study, we showed higher glomerular expression of *Il1b* and its colocalization with PFMs, which showed reduced expression. Moreover, the glomerular expression of *Il1b* correlated with *Tlr8* and tended to correlate with the expression of PFMs (*Nphs1* and *Synpo*). Therefore, these results indicate that factors downstream of *Tlr8*, including *Il1b* and *Il6*, may cause podocyte injury in the obstructed kidney by reducing PFM expression.

The collateral kidneys of young mice also showed GLs and loss of PFMs. This result was confirmed by SEM, which revealed podocyte injury in the collateral kidney. We also found higher levels of serum miR-21 in the ON mouse model. Furthermore, IL1β protein co-localized with PFM. These results indicate that serum miR-21 interacts with TLR8 in the podocytes of the collateral kidney and activates the production of downstream cytokines, which in turn reduces podocyte function and podocyte injury. Podocyte injury was also observed in the obstructed and collateral kidneys of old mice *via* a mechanism similar to that observed in young mice.

**Figure 8:**
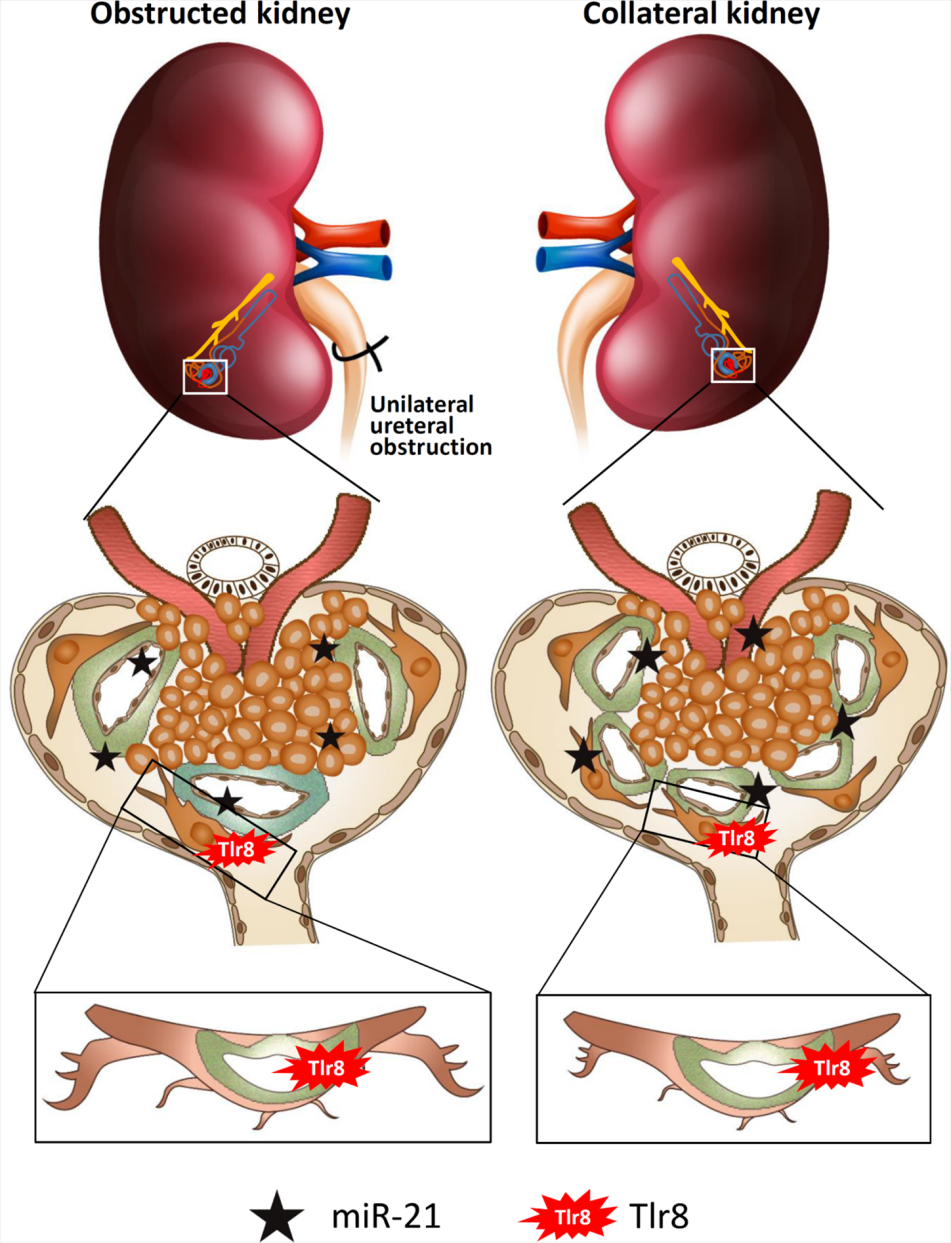
Summary of the study. Elevated levels of glomerular and serum miR-21 due to unilateral obstruction interact with TLR8 in podocytes, which activates the NF-κB-pathway to produce downstream cytokines. Higher levels of cytokines reduce podocyte function markers, resulting in podocyte injury and subsequent development of glomerular lesions. Elevated levels of serum miR-21 activate TLR8 in the podocytes of the collateral kidney and induces glomerular lesions through podocyte injury.

In conclusion (**Figure 8**), glomerular miR-21 expression is elevated following unilateral ureteral obstruction, wherein it interacts with TLR8 in podocytes and induces production of downstream cytokines (*Il1b* and *Il6*) suggesting activation of NF-κB pathway. Higher levels of cytokines reduce PFMs, resulting in podocyte injury and subsequent development of GLs. Elevated levels of serum miR-21 activate TLR8 in the podocytes of the collateral kidney and induce GLs through podocyte injury. Therefore, this study clearly shows GL development in obstructed and collateral kidneys through podocyte injury following *Tlr8* overexpression.

## Data Availability Statement

The original contributions presented in the study are included in the article/supplementary material. Further inquiries can be directed to the corresponding author.

## Ethics Statement

The animal study was reviewed and approved by Institutional Animal Care and Use Committee of the Faculty of Veterinary Medicine, Hokkaido University (approval no. 16-0124).

## Author Contributions

MM: Conceptualization, experimentation, methodology, data analysis, and roles/writing-original draft. OI: Conceptualization, experimentation, and roles/writing—original draft. YE: Conceptualization, investigation, roles/writing—original draft. YK: Conceptualization, supervision, and roles/writing—original draft. All authors contributed to the article and approved the submitted version.

## Funding

This work was supported by a Grant-in-Aid for JSPS Research Fellow from the Japan Society for the Promotion of Science (ID No. 19F19092) and JSPS kakenhi (ID No. 18H02331).

## Conflict of Interest

The authors declare that the research was conducted in the absence of any commercial or financial relationships that could be construed as a potential conflict of interest.
